# Iraqi primary care system in Kurdistan region: providers’ perspectives on problems and opportunities for improvement

**DOI:** 10.1186/1472-698X-12-21

**Published:** 2012-09-27

**Authors:** Nazar P Shabila, Namir G Al-Tawil, Tariq S Al-Hadithi, Egbert Sondorp, Kelsey Vaughan

**Affiliations:** 1 Department of Community Medicine, College of Medicine, Hawler Medical University, Erbil, Iraq; 2 Department of Global Health and Development, London School of Hygiene and Tropical Medicine, London, UK; 3 Department of Development, Policy and Practice, Royal Tropical Institute, Amsterdam, the Netherlands

**Keywords:** Primary care, Care providers, Focus group, Service delivery, Kurdistan region

## Abstract

**Background:**

As part of a comprehensive study on the primary health care system in Iraq, we sought to explore primary care providers’ perspectives about the main problems influencing the provision of primary care services and opportunities to improve the system.

**Methods:**

A qualitative study based on four focus groups involving 40 primary care providers from 12 primary health care centres was conducted in Erbil governorate in the Iraqi Kurdistan region between July and October 2010. A topic guide was used to lead discussions and covered questions on positive aspects of and current problems with the primary care system in addition to the priority needs for its improvement. The discussions were fully transcribed and the qualitative data was analyzed by content analysis, followed by a thematic analysis.

**Results:**

Problems facing the primary care system included inappropriate health service delivery (irrational use of health services, irrational treatment, poor referral system, poor infrastructure and poor hygiene), health workforce challenges (high number of specialists, uneven distribution of the health workforce, rapid turnover, lack of training and educational opportunities and discrepancies in the salary system), shortage in resources (shortage and low quality of medical supplies and shortage in financing), poor information technology and poor leadership/governance. The greatest emphasis was placed on poor organization of health services delivery, particularly the irrational use of health services and the related overcrowding and overload on primary care providers and health facilities. Suggestions for improving the system included application of a family medicine approach and ensuring effective planning and monitoring.

**Conclusions:**

This study has provided a comprehensive understanding of the factors that negatively affect the primary care system in Iraq’s Kurdistan region from the perspective of primary care providers. From their experience, primary care providers have a role in informing the community and policy makers about the main problems affecting this system, though improvements to the health care system must be taken up at the national level and involve other key stakeholders.

## Background

The Iraqi health system significantly deteriorated during the last few decades as a result of wars and economic sanctions. The primary care system did not escape this devastation and continues to suffer from problems common to the health care system in general [1-3].

While the need for re-organizing and restructuring the primary care system in Iraq as part of the overall health system is desperately recognized [2,4], there is limited documented knowledge about the challenges and needs of the primary care system in Iraq, particularly in the Kurdistan region [5]. Availability of such knowledge can help policy makers to better direct appropriate action to improve the primary care system. Given their important role and the power they have in the health care system, obtaining perspectives of primary care providers on the problems and priority needs of the system is a corner stone for any improvement to the system [6,7]. The use of focus groups is an effective technique for gaining a deeper understanding about the problem and exploring health providers’ experience and concerns about health services [8,9]. As part of a comprehensive study on the primary care system in Iraq that targets different actors in the system including decision makers, service providers and service users using different research methods, this study sought to explore primary care providers’ perspectives about the main problems influencing the provision of primary care services in Erbil governorate and opportunities to improve the system.

## Methods

### Subject and setting

Primary care services in Iraq are provided by a network of public primary health care centers (PHCCs) that are of two types: the main PHCCs located in main urban and semi-urban areas and the smaller PHCCs located in rural areas. The main PHCCs are staffed by doctors, dentists, pharmacists, nurses, medical assistants, laboratory technicians and a number of administrative and support staff, while the smaller PHCCs located in rural areas are staffed by non-physician providers [10]. Each center has a manager, usually a physician, and an administrative director, usually a nurse or medical assistant. Primary care providers receive regular fixed salaries from the government. The salary is not related to the number of patients visiting the center or consultation fees generated.

Doctors working in PHCCs are mainly general practitioners. General practitioners in Iraq usually have not received any additional formal training after graduation from medical school, apart from a two year clinical internship and one year working in a remote PHCC or hospital. With the increasing number of specialist doctors in Iraq in recent years and the limited number of positions in hospitals and consultancy centers, more and more specialists are assigned to work in PHCCs [1,2].

The private health care sector provides mainly curative services and consists of a number of relatively small surgical hospitals, a high number of physicians’ clinics and many private pharmacies. The private sector has been growing steadily since the last decade. Although figures on public and private health care spending are not available, the growth of the private sector is evident from the disproportionate increase in the number of private hospitals in Iraq in recent years (from 65 in 2004 to 92 in 2010, i.e. 42%) compared to public hospitals (from 197 in 2004 to 229 in 2010, i.e. 16%) [2,11]. There are also many private clinics run by nurses or medical assistants that directly prescribe and sell most kinds of medicines. In fact, regulations that prohibit selling medicines without a prescription are poorly applied in Iraq. There is also poor regulation of the public-private practice in Iraq and most health care providers work in the public sector in the morning and in the private sector after official working hours [2,12].

### Sampling

A multi-stage stratified sampling technique was used for this study. In the first stage, the main PHCCs in Erbil governorate were stratified according to geographical locations. A random sample of PHCCs was selected from each stratum; four each from Erbil city and the district and sub-district centres in Erbil city suburbs and two each from district and sub-district centres outside Erbil city. In the second stage, a physician, a nurse and/or a medical assistant and an administrator were randomly selected from the list of employees at each PHCC. A total of 40 participants were selected and invited to participate in the focus groups.

### Data collection

Four focus groups were conducted between July and October 2010, each lasting approximately ninety minutes. The first focus group included 12 participants from the four PHCCs in Erbil city. The second included another 12 participants from the four PHCCs in district and sub-district centers in Erbil city suburbs. The third focus group included eight participants from the two PHCCs in district centers outside Erbil city. The fourth focus group included another eight participants from the two PHCCs in sub-district centers outside Erbil city. All focus groups were held in a meeting room or doctor’s office in PHCCs.

Each focus group was facilitated by two researchers, one as moderator and the other as observer. The purpose of the study and the ground rules regarding tape recording of discussions and anonymity of all materials were explained at the beginning of each focus group before obtaining the participants’ consent. The study was reviewed and eventually approved by the Research Ethics Committee of Hawler Medical University.

A topic guide was used to lead discussions and included questions about main problems influencing the provision of primary care services and priority needs for improving the system (Table 1). Each session was concluded when the discussion sufficiently covered the topic and no new information was emerging. At the end of each focus group a debriefing discussion between the moderator and the observer was held.

**Table 1:** **Topic guide for focus group discussions **


1. What are the main positive aspects of the current primary care system?
2. What are the main problems facing the current primary care system?
3. What are the priorities for improving the primary care system?
4. What can be the main barriers to the future improvement of the primary care system?
5. Do you have any additional comments about the primary care system and its improvement?

All discussions were conducted in Kurdish and recorded in full. To assure translation accuracy, audio recordings were transcribed and translated into English. The translation subsequently was verified by an additional native Kurdish speaker fluent in English.

### Data analysis

The translated transcripts were analyzed qualitatively using content analysis, followed by thematic analysis using a framework adapted from the WHO conceptual framework of health system building blocks [13]. Two authors reviewed the transcripts independently, compared notes and reconciled the differences. The condensed meaning units were identified and condensed before abstracting them and labeling them with codes. Emerging coding was used to obtain categories. The categories were further discussed between the two coders for identification and formulation of themes and sub-themes. A greater emphasis was placed on themes and sub-themes repeated by more than one group, initially raised themes and sub-themes, strong feelings, or themes and sub-themes of long discussions. We have included discordant views to highlight differing experiences or perceptions of individuals and groups [8]. Given the manageable length of the four focus groups, no software was used to analyze the data.

## Results

In total 40 primary care providers participated in the focus groups: 12 physicians, 6 nurses, 10 medical assistants and 12 administrators. The participants’ mean ± SD age was 34.4 ± 7.8 years and their median experience in the health system was 7 years (range 1 to 30 years). Details of demographic and professional characteristics of the participants are shown in Table 2. The four focus groups provided a wide representation of views and sufficient saturation. Problems in the primary care system as emerged from the focus groups can be classified under five main themes: 1) inappropriate health services delivery, 2) health workforce challenges, 3) shortage in resources, 4) poor information technology, and 5) poor leadership/governance.

**Table 2:** **Demographic and professional characteristics of participants **

**Characteristic**	**Male**		**Female**		**Total**	
	**No.**	**(%)**	**No.**	**(%)**	**No.**	**(%)**
**Profession**						
Physician	8*	(66.7)	4**	(33.3)	12	(30.0)
Medical assistant	8	(80.0)	2	(20.0)	10	(25.0)
Nurse	4	(66.7)	2	(33.3)	6	(15.0)
Administrator	12	(100.0)	0	(0.0)	12	(30.0)
**Education**						
College	10	(71.4)	4	(28.6)	14	(35.0)
Technical institute	17	(89.5)	2	(10.5)	19	(47.5)
Secondary school	5	(71.4)	2	(28.6)	7	(17.5)

* Six of these 8 physicians were also managers of the PHCC.

** One of these 4 physicians was manager of the PHCC.

### Inappropriate health services delivery

Issues related to health services delivery particularly in terms of organization of these services were the main focus of all focus groups. These issues can be classified under the following subthemes:

### Irrational use of health services

Focus group participants thought that PHCCs suffer from overcrowding which prevents physicians from having adequate time to provide quality care to patients. “*[We see] patients from9:00 to 11:00 a.m.I can [only give]two minutes for each[patient] because of crowdingand because patients arealways in a hurryand impatient to wait”* (F(=female)7, physician). Participants thought that overcrowding is partially the result of patients seeking unnecessary care.

Participants thought the low consultation fees (250 Iraqi Dinars ($0.2)) charged by PHCCs encouraged irrational and repeated visits to PHCCs. They suggested that increasing the fees to 1000 or 2000 Iraqi Dinars per consult might help in reducing many unnecessary visits and lower the irrational use of services. One medical assistant suggested *“the [services can beimproved] by increasing thefees with provision ofbetter care and [sufficient]drugs”* (F8, medical assistant). However, some participants had concerns about introducing higher initial fees that might make some patients, particularly the poor and uneducated, hesitant to visit PHCCs. It was mentioned that some patients may not know the seriousness of their illness or they may even turn to inappropriate health seeking strategies like visiting the private nurse clinics. Therefore, they suggested keeping the initial consultation fees at the current rate and charging additional fees for prescription and further services like laboratory tests and x-rays. “*We cannot increase theuser fees. Even at[this] very low [rate],many patients can not[afford] visiting PHCCs. Itis better to obtainadditional fees for extraservices like investigations [toreduce] unnecessary requests forinvestigations”* (M(=male)27, administrator).

Some participants, particularly physicians, expressed great concerns about unlicensed drug sellers including some private clinics. *“Many medical assistants andnurses have opened clinicswhere they prescribe alltypes of medicines includingthose that can causeaddiction or lead toserious side effects. Withmy respect to theirlong experience, [I think]the medicines they prescribeshould be restricted”* (M7, physician). Another participant noted that these clinics *“have taught people totake [many types] ofdrugs together to get[rapid] relief. During the[flu] epidemic of thisyear, different [combinations] ofinjections and several typesof drugs were providedto patients at theseclinics to [get] immediaterelief”* (M13, medical assistant).

Many participants emphasized the need to adopt a family medicine approach in the primary care system. Under a family medicine model patients would visit only the PHCC in their catchment area and see their own family medicine physician. Each family medicine physician would maintain electronic records for all patients. Participants thought this approach would help control irrational and repeated visits by patients. *“If the system ischanged to a familymedicine system, it willbenefit both the providerand the patient asbetter and more organizedservices will be providedto people and theproviders can interact ina good way withpatients”* (M1, physician). The positive experience of the only family medicine center in Erbil was frequently cited as a model that could be adopted in the primary care system.

### Irrational treatment

The type of treatment provided at the PHCCs was described by some participants as irrational and based primarily on symptomatic treatment. “*Frankly speaking, we providetreatment for symptoms withoutknowing the actual causeof these symptoms”* (M3, physician). They attributed this to time constraints related to the high number of patients and a shortage of medicines and diagnostic facilities. *“We only have someantibiotics and simple analgesics.So when [receiving] apatient, whatever he has,I nearly have writtenthe prescription [when] hestarts talking”* (M12, medical assistant). Furthermore, some respondents reported lacking motivation to take good patient histories and conduct physical examinations. “*We have no motivationin this job andthere are many patients.So we write theprescription immediately and sendthe patient out ofroom” (M14, physician)*.

### Poor referral system

Participants indicated that many patients attend PHCCs only to ask for a referral to a specific hospital or consultation department without having a real reason for the referral. *“Referral is [very common]with patients requesting referralfor even very simpleillnesses. We tell thepatient; please sit downand let [a] physiciansee you [as] youmay not require areferral. [But he insiststhat] he needs togo to [that] particularhospital”* (M19, administrator). Physicians usually resist providing these inappropriate referrals, and participants agreed that provision of better services and increasing the health awareness of the people will help in reducing self-requested referrals. *“If proper health careservices are provided atPHCCs and people becomeaware that most ofthe cases can be[dealt with] at thePHCC, we can controlthese unnecessary referrals”* (M14, physician).

### Poor infrastructure and hygiene

The facilities of most PHCCs were described as old, small and lacking sufficient space to provide health services to the current population. “[*This*] *PHCC was built in1987 when [this residential]area was small [andhad] a small numberof inhabitants. The areahas [grown substantially] and[is now inhabited] bya very large numberof people, but thePHCC [remained] the same”* (M17, administrator). Some participants emphasized the poor sanitary situation in PHCCs particularly in the PHCCs located in Erbil city and attributed this mainly to the lack of sufficient numbers of cleaning staff. “*We have only threecleaning staff. If theywork properly they may[maintain the cleanliness] well,but two of themare [completely busy] withorganizing visitors at entryto [the consultation room]and cannot do anythingelse” (*M11, administrator).

### Health workforce challenges

#### High number of specialists in PHCCs

Participants thought that the PHCCs located in Erbil city are becoming like consultancy units as there are many specialists working in different fields. However, the available facilities and medicines at these PHCCs are very limited. “*[This] PHCC has asurgeon, internist, ophthalmologist, otolaryngologist,radiologist, pediatrician and dentist.We just need tochange the name tobecome a consultancy center.But [the available] facilitiesand medicines are stillsimple analgesics and someantibiotics”* (M5, administrator). Some participants indicated that PHCCs often gradually increase the number of specialists in different fields as the local hospitals are unable to accommodate the growing number of newly graduating specialists. Whilst many participants, particularly administrative directors, medical assistants and nurses, emphasized the importance of having specialists at PHCCs, physicians argued that there is no need to have specialists at PHCCs as general practitioners can deal with most patients and can refer them to specialized centers if required. *“[Within] the Iraqi primarycare system [there isno need] to havespecialists in PHCCs. Thereis a need forhospitals in district orsub-district centers to providespecialist [referral] services”* (M18, physician).

#### Uneven distribution of the health workforce

The uneven distribution of the health workforce in PHCCs, particularly with regards to physicians, skilled nurses and medical assistants, was emphasized by some participants. Specific examples of the shortage of skilled health care workers were in the fields of laboratory and radiology personnel. “*We have now anew x-ray unit butit is still notoperating for not havingx-ray [personnel]. There maybe three or four[in a PHCC] butwe do not haveany [at this] PHCC”* (M1, physician).

#### Rapid turnover of the health workforce

A number of participants emphasized the rapid turnover of the health workforce. They indicated that some personnel ask to be transferred to another PHCC shortly after receiving training in a specific job at their current PHCC. They cite different reasons for requesting the transfer, such as moving home to another area. *“Sometimes, [after] we sendtwo staff members toget training on aspecific [program], they askto be transferred toanother PHCC. [Even if]we do not agree,they manage to gettransferred through personal connectionsand [this will affect]that program in thePHCC”* (M2, administrator).

Physicians indicated that there are no incentives to retain physicians at PHCCs. They also thought that they do not have access to specialty training or postgraduate educational opportunities or benefit from supervision from more senior physicians while working in PHCCs. Furthermore, because of high patient numbers and the resulting short consultation times, as well as a lack of essential facilities and medicines, PHCCs are a challenging working environment. This will eventually result in a rapid turnover of physicians. Physicians usually leave PHCCs to pursue specialty training or postgraduate study. *“If I stay atthe PHCC I willremain [a simple employee]without changing anything inmy life*” (M7, physician). Rapid turnover of physicians is a big concern particularly in PHCCs outside Erbil city as physicians usually stay for six months to one year before being replaced by other physicians as part of the physicians’ internship system. Some of these physicians also hold the position of director of the PHCC. Thus, this rapid turnover similarly affects the management of PHCCs. “[The doctor and the administrative director] *need six months tostart understand each otherand once they understandeach other the doctorwill leave [the PHCC]”* (M21, administrator).

#### Lack of training and education

Focus group participants mentioned a lack of opportunities for professional development and education for primary care providers. The available training courses do not always address the actual needs of the providers. *“There are some trainingcourses but these aremainly theoretical [and] cannotbe applied practically. Ipersonally have participated inmany tuberculosis training programs,but we have notimplemented [this program] sofar”* (F4, medical assistant).

#### Salary discrepancies

Participants had concerns about the extreme discrepancies in the salary system. A newly appointed nurse may receive a significantly lower salary than another nurse who has been in the job longer but has the same responsibilities. “*A [newly appointed] nursegets 150,000 or 200,000Iraqi Dinars. It isnot reasonable that anothernurse with less educationbut longer [years inservices] to [get] asalary of 1,350,000 IraqiDinars, while the youngone works like oreven harder than theold one”* (M4, administrator). They agreed that there should be some difference in salaries but not at the extreme levels that are currently present.

### Shortage in resources

Participants agreed that medicines provided to patients in PHCCs often do not cover a full course treatment. The supplied medicines may be sufficient for one or two days only, even if the treatment is required for five or seven days. Prescribing insufficient quantities of medicines is mainly due to the shortage or unavailability of medicines. “*We see patients [atPHCC] for six hours[each day]. Medicines areavailable in the firsttwo hours only. Thenhalf of the patientswill return [home] withouttreatment or [we are]obliged to prescribe medicinesfrom outside the PHCC”* (M18, physician). Many times patients need to come back for another consultation where they may see a different doctor and receive a completely different treatment regimen depending on that physician’s opinion or availability of drugs. “*If a patient haschest infection, he willreceive few capsules thatwill meet two days’need. Since he isnot going to completea full treatment course,he will [return to]you. [The physician] shouldremember the drug [thatwas] prescribed for himtwo days ago”* (M5, administrator).

Some participants emphasized the poor quality of some drugs. “*Many drugs are ofpoor quality that donot function well [and*] *lack real benefit topatients*. *The poor quality isrelated to both* [*poor*] *quality control and poorstorage conditions* [*that is largely related*] *to lack of continuouselectricity particularly in hotweather*” (M6, administrator). Others reported a shortage in some laboratory materials like blood sugar test strips. “[*This*] *small PHCC* [*that does not*] *have large number ofpatients*, [*receives*] *the same quantity oflaboratory materials* [*that is*] *provided to other PHCCthat are bigger and* [*receive*] *more patients*. *The supplies that wereceive every two monthsmeet the needs oftwo weeks only ifwe use them properly*, *so imagine the situationin the bigger PHCCs*” (M3, physician). Participants from PHCCs outside Erbil city noted the role of sector directorates in facilitating the supply and purchase of materials and medicines. The process of obtaining additional supplies and purchasing additional drugs by PHCCs seemed to be easier in PHCCs outside Erbil city compared to those located in Erbil city. “*The administration here isvery helpful*; *having a sector directoratein the district hadmade a lot offacilitation*. *If we have shortagein some supplies likedentistry needles*, *I can process arequest in half anhour*. *If this* [*is*] *done through Erbil*, *it could take* [*longer*] *time* [*or not*] *done at all*” (M27, administrator).

Some participants strongly emphasized the need for PHCCs to have a petty cash fund to be able to take care of urgent maintenance and procurement requirements. Currently PHCCs must follow the usual procurement procedures through the Directorate of Health for all supply and maintenance requests. “*The Directorate of Healthwill carry out theworks*, *but with* [*considerable*] *delay*. *For example it maytake three months* [*to have a new*] *air*-*conditioner*, *but we* [*will have it*] *at end*” (M1, physician). In the PHCCs outside Erbil city, reimbursement of these expenses is easier when facilitated by sector Directorates, but still advance cash payment was a requirement. “*If there is* [*an*] *urgent need*, *we will do itand ask for reimbursementfrom the sector Directorate*. *Even if there* [*is*] *some delay*, *we will eventually getreimbursed*” (M14, physician).

### Poor use of information technology

Many participants were concerned about the improper use of information technology in PHCCs and limited efforts by health authorities to incorporate this important field in the primary care system. “*We have abolished therole of internet andemail*. [*At*] *the Ministry of Healthin Baghdad we weredoing* [*most of the*] *work through email*. *The work was doneimmediately without the needto send letters orrequests* [*by mail*] *and wait* [*to get a response*]. *Each PHCC should haveits own email andknow how to contacteach unit or department*” (M7, physician). Participants agreed that integration of information technology in the primary care system can have a role in improving the organization of health services, disease reporting and communication with the Directorate of Health. While some PHCCs possess computers, they are not used efficiently. “*Each year many graduatesfrom Erbil computer institutecould be recruited bythe Ministry of Healthand* [*with providing*] *2*–*3 computers* [*to*] *each PHCC a goodinformation system* [*can be*] *established*” (M4, administrator).

### Poor leadership/governance

Participants raised a number of issues related to leadership and governance that adversely affect the primary care system. They strongly emphasized the lack of effective monitoring and evaluation within the primary health care system. For example, there is a large problem related to irregular staff attendance; some staff members fail to show up for work without reason and many others leave early before the end of official working hours. “*There is no effectivemonitoring system*. *Even if there aremeasures taken to changethis system*, *for example controlling staffattendance to PHCCs duringworking hours*, *the change will lastonly few days ifthere is no goodmonitoring and follow upon* [*implementation*] *of the new measures*” (M3, physician). They also emphasized the poor regulations of public-private practice and poor control of problems resulting from primary care providers moonlighting in the private sector. Some participants indicated that providers may intentionally provide poor care in the PHCCs in the morning working hours to encourage patients to visit their private clinics after the official working hours. “*The physician may providegood care to thepatients that visit theprivate clinic but maynot provide that goodcare while working inthe public sector*” (M25, medical assistant).

Many of the problems at the PHCCs that were mentioned in the different themes and sub-themes like uneven distribution of the health workforce, poor professional development, poor infrastructure and hygiene, shortage and poor quality of medicines are also related to poor governance/leadership. For example, the participants strongly emphasized poor distribution of workforce skills. “*Sometimes the PHCC isoverstaffed but there maybe shortage in aspecific field like askilled nurse*. *The directorate of healthsends a person tocover this shortage*, *but they send usa clerk*. *How can we carryout health work witha clerk*?” (M3, physician).

## Discussion

Poor organization of health services delivery in Iraqi PHCCs was also reported by other studies [5,14,15]. Such poor organization, the related overuse of services and unnecessary workload on the primary care providers and primary care facilities can have negative effects on the quality of the provided services, particularly on the provider-patient interaction and communication in addition to consultation length [16]. Poor primary care referral in Iraq with high and inefficient referrals and a high rate of self-requested referrals have been underlined by two other studies [17,18]. In fact, optimal referring processes are crucial for the effectiveness, safety and efficiency of health care [19]. Irrational treatment, which is a newly reported problem in the Iraqi primary care system, and provision of incomplete courses of treatment were strongly and repeatedly emphasized by focus group participants. This may lead to non-compliance with its undesirable impact on clinical outcomes and increased financial burden on society [20].

While the problem of health workforce shortages was a main concern in the Iraqi health system a decade ago [1,2], uneven distribution of human resources for health, particularly of the skilled workforce, is becoming a bigger concern today [1,2,5,21]. The uneven distribution of the health workforce, with doctors or other skilled workers concentrated in main PHCCs and not in smaller PHCCs or in the PHCCs located in city centers and not outside the city centers, is an inefficient allocation of staff and contributes to inequity in health provision [22]. Poor professional development for health care providers has also been reported by other studies from Iraq where poor training of primary care providers and the importance of increasing public investment in this area and reviewing the professional and medical standardization have been emphasized [12,23,24]. This is also a common problem in other post-conflict countries like Serbia where few opportunities for professional development of primary care providers have been reported [25]. While the negative effects of brain drain on the Iraqi health system are well documented [24,26], the effect of rapid turnover of skilled workforce has rarely been reported even though it is an established problem in the primary care system.

Poor governance and leadership in the Iraqi health system has also been emphasized by other studies where the system was described as hospital-based and capital-intensive requiring large quantities of imported medicines, medical supplies and equipment. Other studies have found poor governance and policy processes to be the main concerns of the health system [1,27]. Similarly, reorganization of services and leadership were recognized as the main priorities for reforming the primary care system in Serbia [25].

Several suggestions to improve the primary care system in Iraq that were identified by this study correspond well with other studies from Iraq including application of a family medicine approach [5,12,28], regulation of public-private practice [12] and improving the quantity and quality of medications [5]. Moreover, new priority needs were emerged from this study including establishment of a functioning recording system, increasing the health awareness of the population, provision of incentives to retain staff, integration of health education services in the primary care system and establishment of a strong planning, evaluation and monitoring system at the Directorate of Health and Ministry of Health levels. Increasing user fees to prevent irrational use of services and overcome other problems related to inappropriate health services delivery as outlined in this paper remains a matter of debate.

Most of the problems identified by study participants, such as the uneven distribution and rapid turnover of the health workforce, shortage in resources, lack of information technology and poor planning and monitoring have their roots in the poor leadership and governance of the health system at the national level. While the perspectives of health care providers and managers are important for care improvement and policies formulation, this should be done through proper channels and with full support of the Ministry of Health and not only the Directorates of Health. Therefore, this process should be properly planned and guided by a clear strategy involving all key stakeholders, and not solely based on the opinions of one group of health care providers.

The use of focus group discussions in this study was a new approach in Iraq. Our findings highlight the importance of using qualitative data to facilitate an in-depth and wider understanding of the challenges facing the primary care system [29]. Participants from a range of professions in the primary care setting were included in the study to maximize exploration of different perspectives within a group setting [30,31]. The results, therefore, give a reasonable representation of primary care providers and of different opinions and concerns. Though this variation encouraged interaction and exploration of different perspectives which in many cases led to discordant opinions, we noticed that physicians and administrative directors dominated the discussions. Thus, this study may under-represent the opinion of nurses and medical assistants.

The problems and priority needs of the Iraqi primary care system identified by this study reflect only the perspectives and experience of primary care providers. We recognize that this does not give the entire picture of the problems facing the system and how services need to change as it does not include the perspectives of beneficiaries and decision makers at the Directorate of Health. Nonetheless, these findings are a first step towards improving the system, especially because primary care providers will have an important role in implementing any reform [6,7].

The trustworthiness of this qualitative research can be considered in terms of credibility, dependability and transferability [32]. With regards to credibility, this study had a clear focus and the processes of data collection and analysis have confidently addressed this focus. As the study was carried out over a limited period of time with four focus groups conducted during a three month period, the study should have fair dependability. The chief limitation of qualitative studies remains the inability to generalize findings. However, the results are transferable to populations and contexts similar in characteristics as the data have been carefully collected and analyzed, with the main aim of understanding instead of seeking explanations [33]. Moreover, the focus group climate was open and the respondents willing to share information were free and comfortable to do so, but underestimation cannot be ruled out. The predominance of male providers in the sample might be seen as a limitation. However, the female providers participated actively in the discussions and felt free and comfortable to express their opinions about the primary care system. We could not recognize any clear differences between male and female providers in their perspectives about different aspects of the primary care system.

These findings may provide insight for primary care managers and providers to improve the primary care services in Kurdistan. However, most of the problems described in the focus groups are largely outside the control of managers and providers. The findings may also inform and influence managers at the Directorate of Health and Ministry of Health and other policy makers of the wider contextual issues affecting primary care settings. Further similar research in other governorates of Kurdistan and Iraq could show how generalizable these findings are. Additionally, research is needed on the perspectives of primary care users and the general population.

## Conclusions

This study has provided a comprehensive understanding of the factors that negatively affect the primary care system in Iraqi Kurdistan from the perspective of primary care providers. Primary care providers have a key role in informing the community and policy makers about the main problems affecting this system, and in this way can influence primary care policy. However, a wider effort involving other stakeholders is necessary to develop a clear strategy for improving the primary care system in Iraq.

## Abbreviations

PHCC: Primary health care center.

## Competing interests

The authors declare that they have no competing interests.

## Authors' contributions

NPS, NGAT and ES conceptualized the study. NPS, NGAT and ES participated in designing the study. NPS and NGAT collected the data and carried out data analysis. NPS, TSAH and ES drafted and finalized the manuscript. TSAH, ES and KV extensively reviewed and edited the manuscript. All authors read and approved the final manuscript.

## Pre-publication history

The pre-publication history for this paper can be accessed here:

http://www.biomedcentral.com/1472-698X/12/21/prepub
